# Liquid biopsy and multi-analyte testing guided treatment of *HER2* positive periampullary adenocarcinoma with durable complete response after trastuzumab based therapy

**DOI:** 10.18632/oncotarget.27793

**Published:** 2020-11-10

**Authors:** Rajnish Nagarkar, Darshana Patil, Sewanti Limaye, Pradip Devhare, Ashwini Ghaisas, Navin Srivastava, Sachin Apurwa, Sanket Patil, Jinumary John, Zarrine Raazi, Aditya Shreenivas, Janani Sambath, Ajay Srinivasan, Prashant Kumar, Dadasaheb Akolkar, Rajan Datar

**Affiliations:** ^1^HCG Manavata Cancer Centre, Nasik, Maharashtra, India; ^2^Datar Cancer Genetics Limited, Nasik, Maharashtra, India; ^3^Kokilaben Dhirubhai Ambani Hospital and Medical Research Institute, Mumbai, Maharashtra, India; ^4^Department of Medical Oncology, Medical College Wisconsin, WI, USA; ^5^Institute of Bioinformatics, International Technology Park, Bangalore, Karnataka, India; ^6^Manipal Academy of Higher Education, Manipal, Karnataka, India

**Keywords:** periampullary adenocarcinoma, ETA, HER2, trastuzumab

## Abstract

Periampullary adenocarcinomas are rare neoplasm that originates from the pancreatic head, the ampulla of vater, the distal bile duct or the duodenum. Surgical resection followed by adjuvant therapy is considered as the standard of care treatment for these carcinomas. Despite several advances in diagnostics and therapeutics, only 5% of these patients have an overall survival of five years or more. Currently, there is a dearth of viable therapeutic targets for this disease. The role of HER2 in cancer biology has been studied extensively in several tumour subtypes, and HER2 based targeted therapies have shown to have therapeutic benefits on different cancers. In this case report, we present a case of HER2 positive distal common bile duct carcinoma – a subtype of periampullary carcinoma with multiple relapses where multi-analyte testing with Encyclopedic Tumor Analysis (ETA) (Exacta^®^) identified amplification and over expression of HER2 gene which was used as a potential target to treat the patient with trastuzumab. Synchronous *in vitro* chemosensitivity profiling on Circulating Tumor Asscociated Cells (C-TACs) isolated from blood aided us to design the personalized chemotherapeutic regimen with cyclophosphamide and methotrexate. The combination of trastuzumab with cyclophosphamide and methotrexate yielded excellent treatment response with the patient remaining in complete response till the last follow-up. Our study suggests HER2 directed therapy as a potent pathway for treatment in the subset of HER-2 amplified distal common bile duct carcinomas.

## INTRODUCTION

Periampullary adenocarcinoma has two different morphological types with different prognosis and treatment modality. Pancreatobiliary type (PB type) includes pancreatic cancer, distal common bile duct (CBD) cancer, and some of the ampullary carcinomas and is associated with poor prognosis. Intestinal type (I type) periampullary adenocarcinoma include duodenal carcinoma and some of the ampullary carcinomas and have better prognosis [1–3]. However, 12% of patients show mixed subtype with both intestinal and pancreatobiliary differentiation [4]. Pancreaticoduodenectomy (PD), often known as the Whipple procedure is the standard of care for resectable pancreatic cancer and premalignant lesions in the periampullary region. However, the 5-year postresection survival rate of pancreatobiliary tumors is 20% [5]. It has been reported that after resection 39–67% of patients will have a recurrence with intrahepatic and local recurrence [6].

Due to the high recurrence rates and poor outcomes of these carcinomas, adjuvant therapy, including chemotherapy, radiotherapy and chemoradiotherapy, should be considered. To our knowledge, the ideal sequence and combination of adjuvant chemotherapy or radiotherapy after resection of these tumors remains unclear and is an area of active research.

Postoperative adjuvant radiation therapy with and without concomitant chemotherapy also has limited benefit with frequent relapses and high mortality [7]. Therefore, novel approaches are needed in the treatment of relapsed metastatic periampullary carcinomas to help improve survival. HER2, (Human epithelial growth factor receptor 2), a family of tyrosine kinase receptor are shown to be overexpressed in several human cancers [8]. HER2 mediated signaling is essential for many cellular processes such as cell survival, proliferation and growth [9]. HER2 gene amplification has been reported in pancreatic cancer and has been associated with shorter survival rate [10–12]. Role of HER2 targeted therapies is well established in breast cancer and gastric cancers [13, 14], and its use is currently being explored in other cancer subtypes also. However, the efficacy of HER2 targeted therapies in periampullary cancers is still emerging [5, 15].

Here, we report a case of distal CBD subtype of periampullary carcinoma who has had excellent durable response after HER2 targeted therapy in combination with chemotherapy designed based on Encyclopedic Tumor Analysis (ETA) (Exacta^®^) [16]. Encyclopedic Tumor Analysis (ETA) (Exacta^®^) offers integrative multi-analyte investigations of the tumor which captures in depth information about the multi-layered tumor interactome. Patient can be guided for label-and organ-agnostic, personalized multi-drug therapy recommendations based on inputs from multiple molecular biomarkers as well as *in vitro* chemosensitivity testing on viable tumor cells.

## CASE PRESENTATION

**Figure 1:**
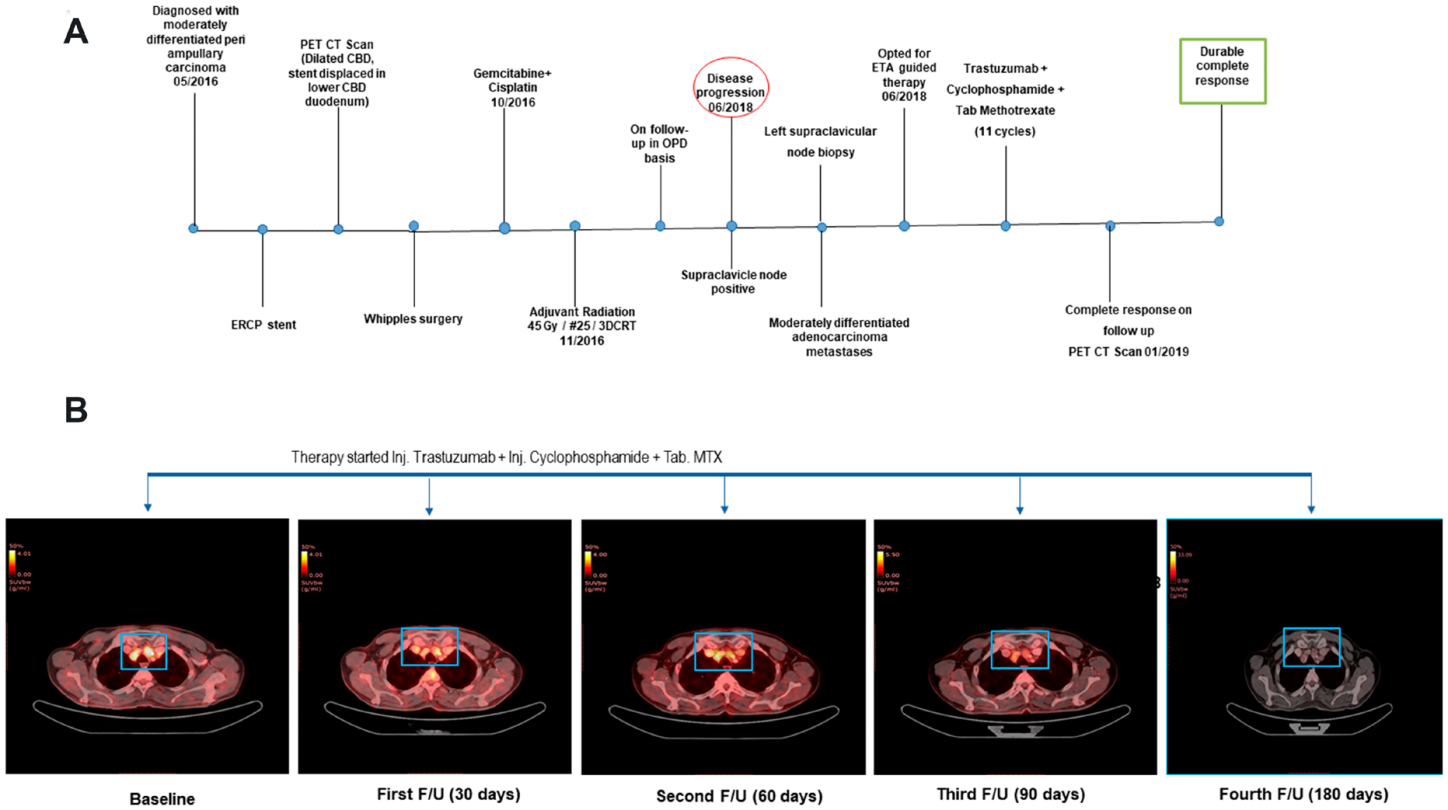
(**A**) The clinical timeline of the 55-year-old male diagnosed with distal CBD type of periampullary carcinoma. (**B**) Transverse sections of representative PET-CT scan images before treatment (Baseline) and after start of Cyclophosphamide + Methotrexate + Trastuzumab regimen depicted complete response.

A 55 years-old male was diagnosed with moderately differentiated periampullary carcinoma (pT3N0). The MRCP images showed defect in distal common bile duct (CBD) causing obstruction of lumen with subsequent dilation of proximal CBD and intra hepatic biliary dilation. The patient underwent ERCP guided stent placement and the ERCP brush cytology was suggestive of differentiated periampullary adenocarcinoma grade II. The patient underwent Whipples surgery after PET CT scan images revealed that the stent was displaced in lower CBD and duodenum. Postoperatively the patient received adjuvant radiation of 45 Gy/ #25 /3DCRT which were followed by 2 cycles of adjuvant chemotherapy with cisplatin and gemcitabine (Figure 1A).

Despite receiving adjuvant treatments, he was found to have disease progression after ~1.6 years of diagnosis, with metastases to the periportal, paraesophageal and left supraclavicular lymph node, which was confirmed by a biopsy of the left supraclavicular lymph node. At the time of disease progression, the patient opted for multi-analyte testing with Encyclopedic Tumor Analysis (ETA) (Exacta^®^) which included next generation sequencing analysis that was carried out on the FFPE tumor tissue DNA, transcriptomic analysis, and chemosensitivity assay which was performed on circulating tumor associated cells (C-TACs) isolated from peripheral blood sample [17]. This multi-analysis identified amplification and over expression of HER2 gene which was used as a potential target to design a treatment regimen for the patient with trastuzumab in combination with chemotherapy agents Methotrexate and Cyclophosphamide (Table 1). The patient was treated with trastuzumab (318 mg), cyclophosphamide (750 mg) and methotrexate (1.5 gm) at 21 days cycle based on the Exacta^®^ analysis. The overview of the therapy procedure is summarised in Figure 1A. Figure 1B delineates the clinical outcome in terms of disease response by representative PET- CT scan images of pre- and post-treatment. Before therapy, a baseline PET-CT scan was done to document the disease status, in terms of increased metabolic activity and size of pelvic, multiple mediastinal, supraclavicular and left cervical lymph nodes. After the start of ETA guided therapy, the metastatic lesions depicted in Figure 1B were almost resolved after 90 days follow-up PET-CT scan compared to the baseline scan.

**Table 1:** Encyclopedic tumor analysis (ETA) findings

Sample Type Received	Gene Name	Molecular alteration
(left supraclavicular node biopsy)	TP53 p. C141R	SNV (32% MAF)
	TP53 p. R158C	SNV (2.2% MAF)
	CDKN2A p. G122D	SNV (2.9% MAF)
	ABL1 p. T315I	SNV (2.1% MAF)
	SYNE1 p. K7182fs*17	SNV (4.6% MAF)
	CDK12, PGAP3, ERBB2 (17q12 - 17q12)	CNV (18 gain)
	CCNE1 (19q12 - 19q12)	CNV (14 gain)
	AR (IHC)	Negative
Blood	Chemo-sensitivity on C-TACs	Cyclophosphamide 38% and Methotrexate 44% CD

MAF – Mutant allele frequency; SNV – Single nucleotide variation; CNV – copy number variation; DGE – Differential gene expression; C-TACs – Circulating Tumor Associated cells; CD – Cell death.

**Figure 2:**
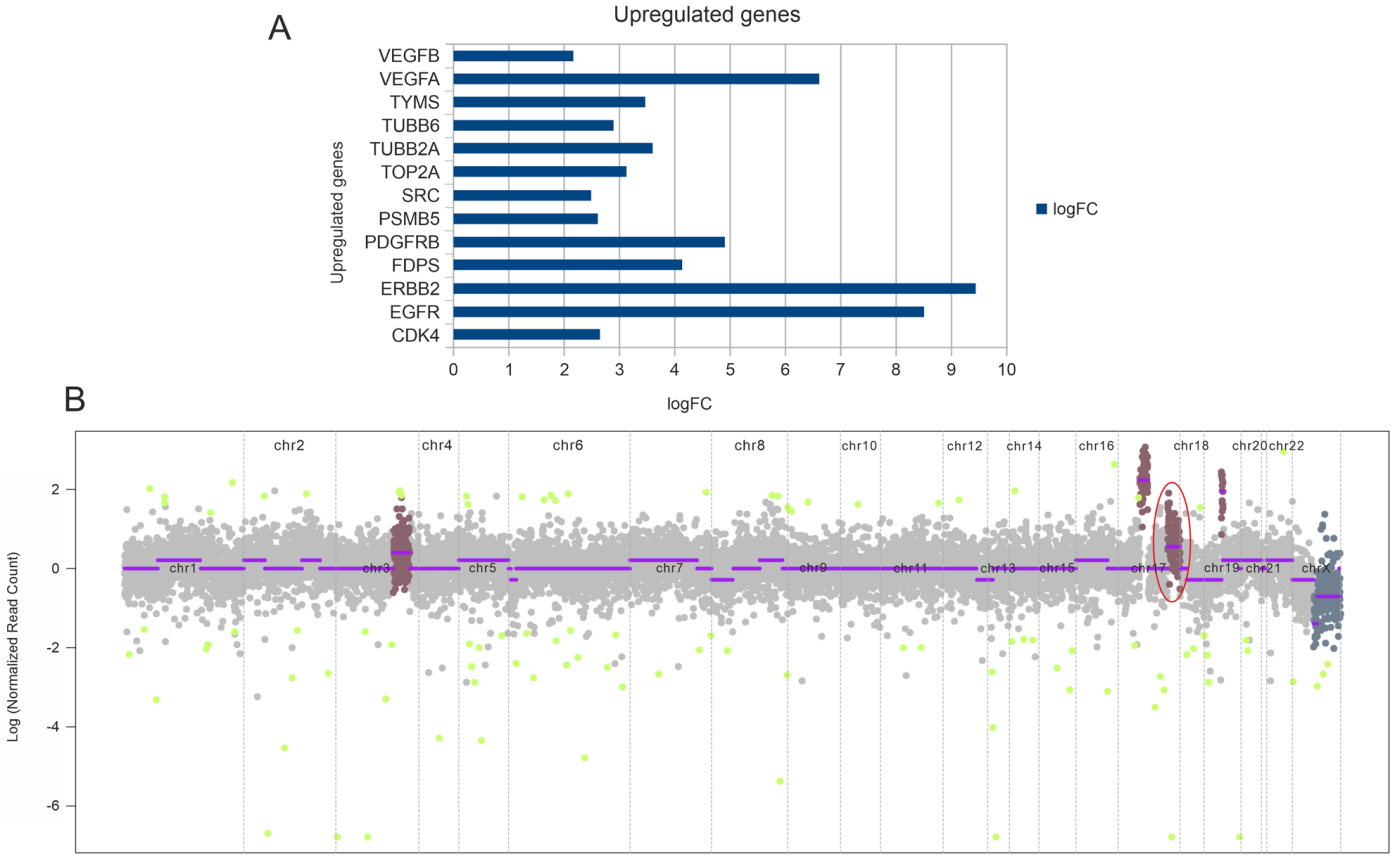
(**A**) Transcriptome analysis depicting upregulated genes based on the fold change values. (**B**) CNV plot depicting copy number amplification. Highlighted is the amplification of *CDK12, PGAP3, and ERBB2* in chromosome 17.

Next generation sequencing analysis was carried out on the FFPE tumor tissue DNA. Tumor mutation analysis detected mutations in genes TP53 (p. C141R), CDKN2A (p. G122D), SYNE1 (p. K7182fs*17) (Table 1). The gene expression analysis showed the expression of 8183 genes in which 3109 genes were differentially regulated. Out of 3109 genes 1374 genes were upregulated and 1735 genes were downregulated (data not shown). Representative up-regulated genes are depicted in Figure 2A. The copy number analysis revealed 18 copy number gain in chromosome 17 (*CDK12, PGAP3, ERBB2)* while 3 copy number gain in chromosome 3 (*RNF213, EPHB1, PIK3CB, FOXL2, ATR)* and 14 copy number gain in chromosome 14 (*CCNE1*) (Table 1 and Figure 2B).

Patient received 11 cycles for 7.7 months of the combination therapy of Cyclophosphamide + Methotrexate + Trastuzumab. ETA produced a good clinical output as there was no disease at the anastomotic site or active distant metastases was not seen. There were no significant therapy related AEs, and the patient experienced transient mild/moderate fatigue, Nausea which were managed clinically. Overall, with complete response patient achieved PFS of 6.9 months and OS of 20.3 months.

## DISCUSSION

The periampullary adenocarcinoma (PAC) is a heterogeneous group of neoplasm arising from the head of the pancreas, the distal common bile duct, and the duodenum. Pancreaticoduodenectomy is the standard clinical management for this disease [18]. The postoperative clinical management options are not standardized due to the rarity of the disease. Gemcitabine-based regimen and fluorouracil-based regimen are mostly used as postoperative treatment options [19–22]. In the present case, after the Whipple’s procedure the patient was given a combination of gemcitabine and cisplatin for 2 cycles followed by adjuvant radiation. However, the patient developed progressive disease after 5 months of treatment completion. The patient was offered multi-analyte testing guided treatment based on ETA approach. It is well known that the histological subtype and genetic signature of the patient may influence the prognosis and treatment response and investigation of molecular profile of the patient could provide opportunities to design personalized treatment regimen for better prognosis and survival.

The NGS analysis of the tumor resected from the patient showed amplification and deletion of many genes and revealed HER2 gene amplification with 18 copy number gain in chromosome 17. The gene expression analysis also showed over expression of HER2 genes with high fold change. Epithelial growth factor receptors (HER1 (EGFR), HER2 (ERBB2), HER3 (ERBB3), and HER4 (ERBB4)) are one of the commonly overexpressed tyrosine kinase receptor families in several cancer types. Approximately 25% of invasive breast cancer patients are characterized by the overexpression of the HER2 receptor and/or HER2 gene amplification with poor prognosis [23, 24]. HER2 is effectively used as a predictive biomarker for anti-HER2 targeted therapy in breast cancer, gastric cancer. Trastuzumab, a recombinant humanized anti-HER2 monoclonal antibody, combined with chemotherapeutic agents has showed improved overall survival in HER2 positive advanced or metastasized gastric or breast cancer patients [25, 26]. Various studies have demonstrated the amplification or overexpression of HER2 in 0–23% of periampullary adenocarcinoma patients [4, 11, 27, 28]. The integration of genomic and transcriptomic data of 60 patients identified CCNE1 and ERBB2 as the putative driver genes in periampullary adenocarcinoma [29]. Data regarding management with targeted therapy with trastuzumab and chemotherapy and other HER 2 directed combinations are emerging for advanced metastatic periampullary carcinoma [30, 31].

In this study, we presented a case of advanced moderately differentiated periampullary adenocarcinoma – the distal CBD subtype where integrative, multi-analyte Exacta^®^ testing helped identify the candidate genes for potential targeted therapy and the chemotherapy regimen with the chemosensitivity testing. Identification of HER-2 amplification led to an opportunity to use trastuzumab based combination therapy that resulted in excellent response. This label-/organ-agnostic treatment approach helped to achieve better clinical outcome in the present case as described previously [16, 32]. HER2 amplification represents potential molecular target for treatment in the subset of HER-2 amplified periampullary carcinomas.
