# Addendum: Liquid biopsy and multi-analyte testing guided treatment of *HER2* positive periampullary adenocarcinoma with durable complete response after trastuzumab based therapy

**DOI:** 10.18632/oncotarget.28296

**Published:** 2022-11-02

**Authors:** Rajnish Nagarkar, Darshana Patil, Sewanti Limaye, Pradip Devhare, Ashwini Ghaisas, Navin Srivastava, Sachin Apurwa, Sanket Patil, Jinumary John, Zarrine Raazi, Aditya Shreenivas, Janani Sambath, Ajay Srinivasan, Prashant Kumar, Dadasaheb Akolkar, Rajan Datar

**Affiliations:** ^1^HCG Manavata Cancer Centre, Nasik, Maharashtra, India; ^2^Datar Cancer Genetics Limited, Nasik, Maharashtra, India; ^3^Kokilaben Dhirubhai Ambani Hospital and Medical Research Institute, Mumbai, Maharashtra, India; ^4^Department of Medical Oncology, Medical College Wisconsin, WI, USA; ^5^Institute of Bioinformatics, International Technology Park, Bangalore, Karnataka, India; ^6^Manipal Academy of Higher Education, Manipal, Karnataka, India

**This article has an addendum:** The patient has previously consented to publication of de-identified findings.

Original article: Oncotarget. 2020; 11:4195–4200. 4195-4200. https://doi.org/10.18632/oncotarget.27793

